# Mathematical optimization of frost resistant crop production to ensure food supply during a nuclear winter catastrophe

**DOI:** 10.1038/s41598-023-35354-7

**Published:** 2023-05-22

**Authors:** Nick Wilson, Ben Payne, Matt Boyd

**Affiliations:** 1 University of Otago, Wellington, New Zealand; 2 Massey University, Wellington, New Zealand; 3 Adapt Research Ltd, Reefton, New Zealand

**Keywords:** Climate sciences, Environmental social sciences

## Abstract

This study aimed to estimate the optimal mix of frost resistant crops and land area needed to provide basic nutrition during various nuclear winter scenarios for New Zealand (NZ), a temperate island nation. It used linear programming to minimize land area required for cropping while producing enough food to achieve dietary energy and protein requirements for the whole population. The potential agricultural impacts of three nuclear winter scenarios on NZ, were sourced from the literature. The optimized combinations of frost resistant crops that were found to feed the entire population were, in descending order: wheat and carrots; sugar beet; oats; onions and carrots; cabbage and barley; canola and cabbage; linseed and parsnip; rye and lupins; swede and field beans; and cauliflower. But in terms of current production levels of these frost resistant crops in NZ, there would be a 26% shortfall for the “war without a nuclear winter” scenario and a 71% shortfall for the severe nuclear winter scenario (150 Tg of soot in the stratosphere with a 61% decline in crop yields). In conclusion, at current production levels, frost resistant food crops could not feed all NZ citizens following a nuclear war. There is a need for the NZ Government to conduct a detailed pre-war analysis on how these shortfalls are best addressed. For example, by: increased pre-war production of these crops and/or post-war scalability; growing enough *frost sensitive* crops (i.e., in greenhouses or the warmest parts of the country); and/or ensuring continuing production of food derived from livestock fed on frost resistant grasses.

## Introduction

The risk of nuclear war may have increased through 2022–2023 as the result of changes in the global geopolitical situation. In particular there is Russia’s invasion of Ukraine in 2022 and the associated threats to use nuclear weapons by its leadership^1^. There has also been an erosion of a key nuclear weapon treaty^2^ and ongoing expansion of some nuclear arsenals (e.g., those of the United Kingdom [UK]^3^, China^4^, and Pakistan^5^). Furthermore, there is the continuing modernization of various nuclear arsenals (e.g., in the United States [US]^6^, France^7^, Russia^8^, India^9^, and North Korea^10^). Taken together, such developments might increase the perceived utility of these weapons in war fighting, and therefore the risk of actual use in war. As a result, previous estimates for the annual probability of inadvertent nuclear war, such as being around 1%^11^, or in the 0.3% to 3% range for all types of nuclear war^12^, may well now be underestimates of the risk of nuclear war.

Climate modeling studies suggest that a nuclear war which generated a nuclear winter (that blocked sunlight and reduced crop production) could potentially be catastrophic^13–17^. “More than 5 billion [people] could die from a war between the United States and Russia” according to one of these studies^16^. This study also estimated persisting impacts on crop production that lasted at least 10 years. These terrible consequences highlight the urgent need to reduce international tensions between states, making nuclear weapons less usable in conflict, and taking verifiable graduated steps towards nuclear disarmament. Nevertheless, such preventive efforts may still fail and so prudent nations that are unlikely to be directly attacked in a nuclear war, should consider planning to maximize their survival chances.

These nuclear winter modeling studies suggest that Southern Hemisphere island nations may experience relatively less severe nuclear winter impacts. Similarly, Southern Hemisphere islands may also be less impacted in a “volcanic winter”, at least based on the impact of one large historical eruption (i.e., Mt Tambora)^18^. For one of these islands, New Zealand, a severe nuclear winter could lower temperatures by up to 5 °C^13^. Modeling of various nuclear winter scenarios on crop production suggest reduced crop yields in the range of 8% to 61%^16^ for New Zealand (see Table 1 for extra details). In some parts of this country, crops may have delayed maturation (e.g., a 3 °C decline in temperature would delay the maturity of wheat crops in the Canterbury region by about 40 days^19^). But in more southern parts of the country full maturation of grain crops may not be possible (e.g., for the Southland region with a 3 °C decline in temperature^19^).

**Table 1:** Four nuclear war and nuclear winter scenarios considered in this study (adapted from:^20^).

Nuclear war and winter scenario	Scale of impact of a nuclear winter	Estimated impact on agricultural production in New Zealand
**No nuclear winter,** following a nuclear war in the Northern Hemisphere (Scenario: NW0)	The war in this scenario is assumed to result in no nuclear winter impacts (reflecting persisting uncertainties in the modeling literature^30^)	Zero from no significant changes to sunlight, temperature, precipitation, and ozone levels
**Some nuclear winter,** following a regional nuclear war (e.g., between India and Pakistan) (NW1)	We used the lower end impact (5 teragram [Tg]) of the estimated 5 to 47 Tg range of stratospheric loading of soot from a nuclear war. This is from a major modeling study published in *Nature Food* in 2022 by Xia et al.^16^	An 8% reduction in major food crops and marine fish* ^16^
**Severe nuclear winter,** following a major Northern Hemisphere war (NW2)	We used the impacts from a New Zealand Planning Council study of a 5000 to 6000 megatonne war in July (Northern Hemisphere summer)^31^. This was assumed to result in a spring temperature reduction in New Zealand of 3 °C, a 2 °C reduction in summer and a 1 °C reduction for another 18 months. Although the relatively high megatonnage of this scale of war may not be particularly realistic in the 2020s – it could still represent the impact of a current-era war with attacks on cities and high levels of stratospheric loading of soot (see the next row). It could also represent a war involving China in the 2030s (since it is expanding its arsenal)	A 28% reduction (mid-point in the estimated 19–36% reduction in pasture growth in year one)** ^32^
**Catastrophic nuclear winter,** following a major Northern Hemisphere war (NW3)	We used the highest value (150 Tg) of stratospheric loading of soot used in the work by Xia et al.^16^. This analysis suggests that cropland solar radiation would be lowest between years 1 and 2 post-war, and take at least 10 years to return to normal. Cropland precipitation would be lowest around year 4 post-war, and also take at least 10 years to return to normal	A 61% reduction in major food crops and marine fish* ^16^

*This study by Xia et al. estimated food energy production for New Zealand as part of a global analysis using data for major food crops (maize, rice, soybean and spring wheat) and marine fish averaged in year two. For modeling parsimony, we used these specific reductions for across-the-board food production, even though grass growth for livestock production may be less impacted than crop production. This 8% reduction value from the Xia et al. 5 Tg scenario compares with a 12% reduction for maize and wheat in year 4 (and a 5% reduction average for years 1–5) in an earlier study (i.e., Jägermeyr et al.: Table S3)^15^.

**This New Zealand work^32^ estimated pasture dry matter production impacts from nuclear war for Waikato, Canterbury and Southland regions with reductions in year one ranging from 19 to 36%. For year two the range involved reductions from 11 to 17%. It has also been estimated for New Zealand that a 3 °C decline in temperature would delay the maturity of wheat crops in Canterbury by about 40 days^19^. This would probably not be a major problem but a 20% decline in solar radiation would result in a probable decline in yield of 15%. Also noted was that in Southland a 3 °C decline in temperature might actually prevent maturation of grain crops^19^. The impact of the frost-free period would be highly variable around the country. For example, for Ophir (Central Otago) a 1 °C drop in minimum temperature decreases the frost-free period by around 24 days (from 93 days), while for Tauranga it decreases by over 50 days (from around 350 days, albeit these estimates were from the 1980s)^32^.

For New Zealand we have previously identified that the country has relatively high levels of food self-sufficiency (i.e., food exports alone could provide 3.9 times current dietary energy intakes for all its citizens^20^). But this work also reported that most of these exported foods (by weight) are dairy products such as milk powder that are probably very vulnerable to post-war disruptions. That is, these foods require many imported inputs (e.g., diesel, fertilizer), daily truck/train transport of milk, and complex processing at large dairy factories. Indeed, most agricultural production in New Zealand is dependent on imported inputs (e.g., of seeds, diesel, machinery parts, fertilizer and pesticides). It is also probably vulnerable to various levels of socio-economic collapse of New Zealand society after a nuclear war, which various authors think possible (e.g.,^21–24^). Such collapse could seriously disrupt food production, transport, processing and retailing and a financial system collapse could limit citizens from being able to purchase food^25^.

One approach to building a country’s resilience to the threat of nuclear winter is to particularly focus on frost resistant crops. Such crops would be the ones most likely to survive more severe winters as well as out-of-season frosts that could occur in growing months. The latter occurred during the “volcanic winter” following the eruption of Mt Tambora in 1815. That is, this eruption caused frosts during the growing season in parts of Europe (e.g., in April and September), the US (June and August), and China (July)^26^. These impacts from Tambora’s “volcanic winter” (along with changes to rainfall and storms) reduced crop harvests and resulted in famines in multiple settings around the world^27^.

Frost resistant cereal crops (e.g., winter wheat) also have the advantage of having more time to grow than spring planted versions in potential year-round reduced sunlight conditions. This is because these cereals are planted in the autumn and can take better advantage of the early spring growing season than their spring planted equivalents. Crops like winter wheat also have some tolerance to freeze–thaw cycles in autumn^28^, albeit varying by cultivar. Cold tolerance declines for winter cereals in spring, although: “plants that are still in the vegetative phase have the ability to re-acclimate [to freezing conditions] following de-acclimation, whereas plants in the reproductive phase only have a limited ability to re-acclimate”^29^. Nevertheless, winter cereals typically suffer some yield loss from “winter injury”, with an estimated annual average loss for winter wheat in the US at 7% (and up to 70% + on some occasions)^28^. Even so, crops are typically relatively efficient sources of food energy and are usually much cheaper to produce than dairy products, meat, and fish. They are also less dependent on needing refrigerated transport and additional processing in factories.

Given this background, this study aimed to identify the optimal combinations of frost resistant crops needed to provide enough dietary energy and protein for the whole New Zealand population after a nuclear war with possible nuclear winter impacts.

## Methods

### Nuclear winter scenarios

The range of nuclear winter scenarios considered is shown in Table 1. The diversity of potential impacts of these scenarios reflects differing number of weapons that could be used, their targeting and residual uncertainty concerning the extent of “nuclear winter” type impacts^30^. So we include a “no winter” scenario as well as scenarios with differing levels of soot injection into the stratosphere following nuclear weapon explosions on major cities and infrastructure in the Northern Hemisphere^16^. This soot is assumed to then reduce solar radiation reaching the planet’s surface which then results in lower surface air temperatures as well as reduced precipitation levels, both of which impede food crop production in both hemispheres^16^. In all these scenarios we assumed a worst-case situation of a complete end to New Zealand’s trade with other countries (including Australia) for both exports and imports. This was also the approach taken in previous New Zealand research^20,31^. We also ignored stockpiled food awaiting export that could be diverted to domestic use since this would only be temporary and is predominantly dairy products (e.g., milk powder) and frozen meat.

### Crop selection and data sources

Selection of frost resistant crops for temperate countries was based on the grading in Table 2^33^. For these crops we then used food composition data (dietary energy and protein) and crop yield data to complete the table of data inputs (Table 3). In most cases New Zealand specific data were available, but where not we prioritized the use of relevant Australian data, then North American data, and then European data.

**Table 2:** Relative frost resistance of temperate crop species (source:^33^, with minor adaptations).

Damaging temperature range	General hardiness rating	Food crops and grasses for livestock
+ 5 to 0 °C	Chill and frost sensitive	Tomato, cucumber
0 to − 2 °C	Very frost sensitive	Potato foliage, French beans, maize, melon
− 2 to − 4 °C	Frost sensitive	Fruit blossom, grapevine in bud burst, cereals in ear, cauliflower curds, broccoli spears, asparagus spears, spring peas, spring lettuce
Crops which are included in the analysis of this current study – data permitting		
− 4 to − 7 °C	Moderate frost hardiness	Winter oats*, spring cereals, cauliflower and broccoli leaves, kale*, white and spring cabbage, sugar* and fodder beet*, onions, swede*, spring canola*, winter lupins*, carrots, winter lettuce, parsnips ***Grasses:*** Italian ryegrass, red clover, alfalfa (lucerne)
− 7 to − 10 °C	Reasonable frost hardiness	Winter barley*, winter canola*, winter field beans*, winter linseed*, savoy cabbage, spinach ***Grasses:*** white clover
− 10 to − 15 °C	Good frost hardiness	Winter wheat* ^#^ ***Grasses:*** perennial ryegrass
Colder than − 15 °C	Very frost hardy	Winter rye* ^#^, dormant deciduous plants including dormant grapevine ***Grasses:*** timothy and fescue grasses

*These crops can be used as livestock forage crops, but they can also be used to feed people.

^#^There are some cultivars that are even more cold tolerant (e.g., down to − 24 °C for the winter wheat cultivar Norstar and − 33 °C for the winter rye cultivar Puma^28^).

**Table 3:** Nutritional data and crop yield data used in the analysis.

Food crop (from Table 2)	Dietary energy (kJ per 100 g)	Dietary protein (g per 100 g)	Crop yield in tonnes per hectare [ha] (range)*	Data sources, comments [database code for New Zealand food composition data^34^] (all data are specifically for New Zealand unless otherwise indicated)
Vegetables				
Broccoli	140	3.8	16	*Nutrients:* [X1020], fresh, raw *Yield:* Based on winter planted
Cabbage (white)	108	1.2	68	*Nutrients:* [X1102], green drumhead, fresh, raw *Yield:* Based on winter planted
Cabbage (savoy)	130	1.7	44 (29 to 59)	*Nutrients:* [X1260], fresh, raw *Yield:* Based on the range from a European study indicating 29–31 tonne per ha (t/ha) without fertilizer and 56–59 t/ha with highest fertilization (fresh matter yields)^35^
Carrots	156	0.6	120 (70 to 170)	*Nutrients:* [X1114], fresh, raw *Yield:* Based on “process carrots” (not “table carrots”)
Cauliflower	79	0.8	33	*Nutrients:* [X1128], fresh, raw *Yield:* Based on winter planted, New Zealand data^36^
Field beans	143	2.1	25 (20 to 30)	*Nutrients:* [X1108], green runner or dwarf, seeds with pod, fresh, raw *Yield:* Based on weight in the pod with the assumption that “90% of the pods are removed from the field”, New Zealand data^36^
Kale	168	4.6	15 (12 to 17)	*Nutrients:* [X1163], fresh, raw *Yield:* Based on New Zealand forage crop data and covers “short”, “intermediate” and “giant” cultivars^37^. This yield range is for dry weight and so it is likely that the nutrient energy and protein (as presented in this table) are slightly under-estimated
Lettuce	57	1.5	24 (18 to 30)	*Nutrients:* [X1265], green lettuce, assorted varieties, raw, fresh *Yield:* Based on the range covering winter (18 t/ha) and summer (30 t/ha) lettuce, New Zealand data^36^
Onions	130	1.4	70 (60 to 80)	*Nutrients:* [X1130], brown, fresh, raw *Yield:* Based on “fresh marketable yields” of the Pukekohe long-keeper onion (which are 10–13% bulb dry matter), New Zealand data^36^
Parsnips	235	1.0	25 (20 to 30)	*Nutrients:* [X1097], raw *Yield:* Based on the range from Canadian data^38^
Spinach	75	2.5	20 (15 to 25)	*Nutrients:* [X1045], English, raw *Yield:* New Zealand data^36^
Sugar beet	180	1.6	67	*Nutrients:* US data,^39^ [FDC ID: 169145], beets, raw *Yield:* Based on the European Union (27 country) average for sugar beet in 2020^40^. Yield does not include potentially edible leaves. This European estimate is compatible with an estimate from a 1980s study for the Waikato in New Zealand which estimated a commercial 60–70 t/ha sugar beet yield, with slightly higher yield than fodder beet^41^. We did not use the NZ yield data as this is for sugar beet as a livestock feed (18–22 t/ha dry weight)^42^
Swede	125	0.8	33 (25 to 40)	*Nutrients:* [X1167], peeled, fresh, raw *Yield:* Based on Australian data for crops for human consumption^43^. Of note is that this yield does not include the green tops which can also be eaten by people. New Zealand forage crop data indicate a yield of 12 to 16 t/ha but this is for dry weight^44^
Grains				
Barley	1300	9.2	7.0	*Nutrients:* [E1], wholegrain, raw *Yield:* Based on a 5-year average for New Zealand reported in 2020^45^. Barley is mainly planted in mid-spring in New Zealand^46^
Canola	2686	18.6	3.1	*Nutrients:* Based on animal feed data (wet weight) for Europe^47^. We note some compounds in canola meal can impede uptake of some micronutrients (e.g., glucosinolates and phytates) and so additional processing may be desirable for optimal human nutrition^48^ *Yield:* Based on the European Union (27 country) average for 2020^40^. A New Zealand farm has reported an “unofficial new world record” yield of 6.3 t/ha^49^
Linseed	1890	18.4	2.8 (2 to 3.5)	*Nutrients:* [Q1027], linseed or flaxseed, brown or golden, whole, dried, raw *Yield:* Based on the range from good dryland cropping soils in New Zealand (2–2.5 t/ha) to irrigated soils (3–3.5 t/ha)^50^
Lupins	1550	36.2	2.2	*Nutrients:* US data,^39^ [FDC ID: 172423], mature seeds, raw *Yield:* Based on Western Australia data^51^
Oats	1586	13.2	7.9 (5.7 to 10)	*Nutrients:* Based on US data^39^ [FDC ID: 2343973], raw oats *Yield:* Based on Southland, New Zealand, yields^52^. These yields are much higher than the European Union (27 country) average of 3.3 in 2020^40^
Rye	1230	11.0	4.3	*Nutrients:* [E29], wholegrain flakes, raw *Yield:* Based on the European Union (27 country) average in 2020^40^
Wheat	1400	13.4	9.9	*Nutrients:* [E35], wholegrain, raw, North Island, New Zealand *Yield:* Based on New Zealand data for 2020 (and very similar to a 5-year average)^45^. However, a New Zealand farm has reported a world record of 17 t/ha^53^. An estimated 75% of wheat in New Zealand is planted as “winter wheat” (i.e., autumn planting)^46^
Grass-fed livestock products**				
Cow’s milk	278	3.5	5.4 (milk)	*Nutrients:* [F1086], milk, cow, whole 4% fat, fluid, non-homogenized *Yield:* Using a New Zealand average of 650 kg milk solids per ha (using the latest year for data in Figure 15 in:^54^). Converted back into whole milk in the adjacent column by an adjustment of 8.3 (100/12) given that ~ 88% of milk is water^55^
Lamb	581	20.6	0.104 (meat)	*Nutrients:* [M1150], lamb, forequarter & hindquarter assorted cuts, separable lean, raw *Yield:* Using a New Zealand average of 104 kg meat per ha (using the latest year for data in Figure 17 in:^54^).***
Beef	746	20.0	0.310 (meat)	*Nutrients:* [M1220], Beef, forequarter & hindquarter assorted cuts, separable lean & fat, raw *Yield:* Using a New Zealand average of 310 kg meat per ha (using the latest year for data in Figure 16 in:^54^)

*Yields are typically for the “marketable yield” (not fresh biomass in the field), for one crop planting and for New Zealand^36^, unless details for another country are stated. Where a range is reported, the analyses used the mid-point of the range.

**Yields for these livestock products could be slightly higher if the analysis was expanded to estimate the meat from dairy cows (at the end of their milking lives), and if all edible parts of the animal carcasses were included (e.g., organ meats, the marrow inside of bones, and bone meal).

***In disaster circumstances production of lamb (largely for export markets) could shift to the more efficient production of mutton from mature sheep carcasses. Mature sheep also have the other benefit of providing wool.

### Business-as-usual dietary intakes of the New Zealand population

The estimated dietary energy intake of the entire New Zealand population has previously been estimated at 44.4 billion kJ per day, equivalent to 8686 kJ per person per day^20^. Here we used the same approach to calculate protein intake, as per Table 4.

**Table 4:** Estimated daily protein intake of the total New Zealand population.

Population group	Average estimated daily protein intake in grams (nutrition survey data^56,57^)	Population size (Q4 2021 estimates^58^)	Total per day (tonnes)
Adult men (15 + years)	102	2,041,970	208
Adult women (15 + years)	71	2,105,180	149
Children* (< 15 years) **–** males	62	496,930	31
Children* (< 15 years) **–** females	52	470,720	24
Total	–	5,114,800	413** (equivalent to 81 g/person/day)

*We used the protein intakes for the 5–6-year-old-age-group, which is likely mid-range for the 0–14 year group. That is, the intakes for the < 5 year age-group were not collected in the survey data and for the 11–14 year age-group the intakes were boys: 88 g/d; and girls: 66 g/d^57^.

**This total might be slightly below the ideal for planning purposes since some of the adult survey respondents reported food insecurity, some people may have been dieting to control their weight at the time of the survey, and because of under-estimation of food intakes associated with the use of food diaries^59^. Nevertheless, the intakes for adults in New Zealand are relatively high compared to the dietary recommendations of 64 g/d for men (63% of current intakes) and 46 g/d for women (65% of current intakes) (with recommendations being for the 19–70-year-age-group)^60^.

### Optimization

We aimed to identify the minimal amount of cropping land to provide sufficient frost resistant crops to feed the entire New Zealand population. For the mathematical optimization we used linear programming conducted with Excel Solver (using the “Solver LP” method). The objective function was the minimization of the total cropping land required (ha) and the constraints in the base case were to achieve ≥ 8686 kJ/day of dietary energy per person and ≥ 81 g/day of dietary protein per person. These constraints were modified in various scenarios (see below) and comparisons were made with the total area of crop land used in 2019 in New Zealand (132,717 ha in horticulture and 487,763 ha in grain)^61^. More specifically, the level of current frost resistant crop production was assessed in terms of its capacity to feed the population under the different scenarios.

### Scenarios for nutrition and crop selection

Reduced dietary energy (10% less) and protein (35%) requirements were considered in Scenario A. This was on the basis that many people could probably tolerate slightly lower energy intakes in a disaster situation, and the current protein intakes are relatively high compared to recommended levels (see Table 4 footnotes).

Scenario B assumed 50% of both energy and protein intakes from frost resistant foods and the rest from other food sources. The latter could include:

- Continuing production of some *frost sensitive* crops (e.g., potatoes) that could potentially be grown if there were no out-of-season frosts in warmer parts of the country and/or in greenhouses.
- Continuing production of grass-fed livestock products (e.g., dairy and meat from grass-fed livestock near towns/cities or railway networks). Persisting grass-fed livestock production is highly feasible during a nuclear winter given that all the major pasture grasses grown in New Zealand are frost resistant (e.g., the ryegrass and clover^62^ included in Table 2).
- Continuing production of frost resistant vegetables and poultry from home gardens, urban community gardens, and Māori community gardens^63^.

## Results

The optimized combination of frost resistant crops in the base case was a combination of wheat (97% of the required cropping area) and carrots for the remainder (Table 5). This combination was estimated to be able to provide all the dietary energy and protein for the New Zealand population using 116,000 ha of land, which is equivalent to 19% of the current cropping land used for all crops (grains and horticulture). The next most efficient crop/s were, in descending order: sugar beet; oats; onions and carrots; cabbage and barley; canola and cabbage; linseed and parsnip; rye and lupins; swede and field beans; and cauliflower (Table 5).

**Table 5:** Top ten crops/crop combinations and land required for optimized provision of dietary energy and protein per unit cropping area (ha) in the “no nuclear winter” scenario (Scenario NW0).

Prioritized frost resistant crops/crop combinations	Total cropping area required (ha)*	Equivalent % of current area in New Zealand used in cropping	Current New Zealand production (ha)
1) Wheat (96.8% of area), carrots (3.2% of area)	116,000	18.6%	*Wheat:* 43,500 ha in 2021 ^64^ *Carrots:* 1851 ha in 2017 ^65^
2) Sugar beet (100% of area); when excluding wheat	141,000	22.7%	*Sugar beet:* The amount currently grown was not identified (it is grown as a livestock feed, see Table 3)
3) Oats (100% of area); when excluding wheat and sugar beet	145,000	23.4%	*Oats:* 5500 ha in 2022 ^66^. It has been higher in previous years (e.g., 8900 ha in 2010)
4) Onions (88.6% of area), carrots (11.4% of area); when excluding wheat, sugar beet and oats	159,000	25.6%	*Onions:* 5588 ha in 2020 ^67^ *Carrots:* 1851 ha in 2017 ^65^
5) Cabbage (white) (60.9% of area), barley (39.1% of area); when excluding all the crops in the rows above	202,000	32.5%	*Cabbage*: Data are combined with broccoli and cauliflower: 3632 ha in 2017 ^65^ *Barley:* 44,200 ha in 2021 ^64^
6) Canola (51.8% of area), cabbage (savoy) (48.2% of area); when excluding all the crops in the rows above	229,000	37.0%	*Canola:* Area not identified but reported production was 2200 tonnes in 2020 ^68^ *Cabbage:* Data are combined with broccoli and cauliflower: 3632 ha in 2017 ^65^
7) Linseed (93.2% of area), parsnip (6.8% of area); when excluding all the crops in the rows above	304,000	49.0%	*Linseed:* Estimated at 1910 ha in 2019 ^69^ *Parsnip:* Data not identified
8) Rye (95.9% of area), lupins (4.1% of area); when excluding all the crops in the rows above	311,000	50.1%	*Rye:* Data not identified *Lupins:* Data not identified
9) Swede (61.4% of area), field beans (38.6% of area); when excluding all the crops in the rows above	414,000	66.8%	*Swede:* Area not identified. A closely related crop (turnip) had reported production of 75,000 tonnes in 2020 ^70^ *Field beans:* Data are combined with peas: 4705 ha in 2017 data^65^
10) Cauliflower (100% of area); when excluding all the crops in the rows above	622,000	100.2%	*Cauliflower:* Data are combined with broccoli and cabbage: 3632 ha in 2017 ^65^

*For most of these crop/crop combinations, an exact optimization solution could be obtained (i.e., exactly the specified amounts of both energy and protein for the whole population). The exceptions involved excess energy available once the required amount of protein was achieved (i.e., the options involving sugar beet [5% excess] and oats [12%]). Similarly, for there being excess protein once energy requirements were met in one case (the cauliflower option: 9% excess).

The least efficient use of land to produce dietary energy was grass-fed lamb which was 310 times less productive in dietary energy per ha than carrots (beef was the next most inefficient). Milk from grass-fed dairy cows was also relatively inefficient, but superior to two crops (spinach and lettuce) for dietary energy per ha. The least efficient source of protein was also lamb which was 62 times less productive in dietary protein per ha than wheat (beef then milk, were the next most inefficient).

Table 6 maps the base case and two nutrition scenarios (A, B) against the four nuclear war scenarios (NW0, NW1, NW2, NW3). For base case nutrition the need for cropping land to grow wheat and carrots was up to 48% of current cropping land for the most severe nuclear winter scenario (NW3) considered (at 61% reduced agricultural productivity).

**Table 6:** Optimized crop/s for providing dietary energy and protein while minimizing land use (ha) for cropping according to different scenarios (% of current cropping land).

Base case and scenarios	Optimized crops [% of area]	Nuclear war but otherwise business-as-usual agricultural productivity (Scenario NW0)	8% reduction in agricultural productivity from nuclear winter (Scenario NW1)	28% reduction in agricultural productivity from nuclear winter (Scenario NW2)	61% reduction in agricultural productivity from nuclear winter (Scenario NW3)
***Base case:*** Meeting both dietary energy and protein requirements for the whole New Zealand population (current levels)	Wheat [96.8%], carrots [3.2%]	116,000 ha (18.6%)	126,000 ha (20.3%)	161,000 ha (25.9%)	297,000 ha (47.8%)
***Scenario A:*** As per the base case but using plausibly acceptable lower levels of dietary energy (10% less) and protein (35% less) due to relatively high intakes in the current era (see Table 4 for currently high protein intakes)	Wheat [58.7%], carrots [41.3%]	92,000 ha (14.8%)	100,000 ha (16.1%)	128,000 ha (20.6%)	236,000 ha (38.0%)
***Scenario B:*** As per the base case but assuming only 50% of both energy and protein intakes from frost resistant crops (assuming the rest of the food is obtained from additional sources of frost sensitive crops [e.g., in green houses] and grass-fed livestock products – see *Methods*)	Wheat [98.3%], carrots [1.7%]	58,200 ha (9.4%)	63,200 ha (10.2%)	80,800 ha (13.0%)	149,000 ha (24.0%)

For Scenario A, with plausibly acceptable lower levels of dietary energy and protein intakes, the cropping land required to grow wheat and carrots ranged from 15% of the current level (for war with no nuclear winter [NW0]), up to 38% of the current level (for the most severe nuclear winter scenario; Table 5).

In Scenario B, where it was assumed that half of all dietary energy and protein comes from other sources (e.g., from some *frost sensitive* crops and grass-fed livestock) then even less crop land would be required. That is, it would range from 9 to 24% of current cropping land (for the no nuclear winter and most severe nuclear winter scenarios respectively).

In the base case, current levels of frost resistant crop production in New Zealand were estimated to be capable of providing 74% of the dietary energy of the population in the no nuclear winter scenario (i.e., leaving a 26% shortfall, Table 7). But this level of provision was only 29% for the severe nuclear winter scenario (i.e., leaving a 71% shortfall). When considering the possibility of 50% of food from other sources (e.g., *frost sensitive* crops in greenhouses and grass-fed livestock) the current levels of frost resistant crop production could provide an excess of energy in all scenarios except for a shortfall in the severe nuclear winter scenario (i.e., with only 58.5% of the needed production occurring, Table 7).

**Table 7:** Gap analysis for current levels of frost resistant crop production in New Zealand, relative to dietary energy requirements for the whole New Zealand population in various scenarios*

Base case and nutrition scenarios	Nuclear war but otherwise business-as-usual agricultural productivity (Scenario NW0)	8% reduction in agricultural productivity from nuclear winter (Scenario NW1)	28% reduction in agricultural productivity from nuclear winter (Scenario NW2)	61% reduction in agricultural productivity from nuclear winter (Scenario NW3)
***Base case:*** Meeting dietary energy requirements for the whole New Zealand population (current levels) from frost resistant crops	74.4%	68.5%	53.7%	29.3%
***Scenario A:*** As per the base case above but using plausibly acceptable lower levels of dietary energy (10% less)	82.7%	76.1%	59.7%	32.5%
***Scenario B:*** As per the base case but assuming only 50% of both dietary energy and protein intakes from frost resistant crops (assuming the rest of the food is obtained additional sources of frost sensitive crops and grass-fed livestock products – see *Methods*)	149% (oversupply)	137% (oversupply)	107% (oversupply)	58.5%

*Based on the crops and yield data in Table 5, for all frost resistant crops together, except those with missing production data for New Zealand: sugar beet, rye, and swede. Given that sugar beet (or the similar fodder beet) production is probably non-trivial in New Zealand, the results in this table are probably underestimates.

## Discussion

### Main findings and interpretation

This study has found that the theoretically most optimal single frost resistant crop for New Zealand from a combined dietary energy and protein perspective is wheat. It is already grown in New Zealand, including at world-leading yield levels^53^, albeit with dependence on imported diesel, fertilizer and pesticides. Wheat also has the advantages of not requiring refrigeration, being relatively energy dense (which reduces food transportation costs), and excess can be fed to livestock (e.g., poultry for egg production). But the other frost resistant crops favored in the optimization analysis in Table 5 also have some of these same advantages of less complex inputs and less complex processing requirements compared to New Zealand’s current major food exports of dairy products and frozen meat.

Frost resistant crops other than wheat could also have a comparative productivity advantage in specific locations where they are currently grown in New Zealand (e.g., onions and carrots in Pukekohe; oats, barley, linseed and rye in Canterbury etc.). Vegetable crops also have the added advantage of not necessarily requiring further processing (relative to grains that typically requiring milling, although un-milled grain can still be cooked and eaten). Furthermore, most vegetables can potentially be harvested before full size if nuclear winter effects were more severe than anticipated during summer months (unlike grains which need to reach full maturity).

Another key finding was that at current production levels of frost resistant crops, there would be shortfalls in dietary energy (of 26% for the no nuclear winter scenario and 71% for the severe nuclear winter scenario; Table 7). Nevertheless, such deficits could potentially be addressed by some ongoing production of the food sources considered in Scenario B. That is, attempting to maintain some production of *frost sensitive* crops in green houses or if there were no out-of-season frosts in summer months in warmer parts of the country. This might be particularly feasible for crops like potatoes (which can survive some frost damage to foliage) than for fruit (e.g., apples, avocados and kiwifruit). Another option would be maintaining grass-fed livestock production on hill country near towns and cities and along railway networks (especially the electrified part of the North Island railway network). If biodiesel for trucking was unavailable, then sheep and cattle could be “walked” via “cattle drives” to railway depots or directly to abattoirs in nearby towns and cities.

### Comparability with other studies

Other work considering sunlight-blocking catastrophes (for the US)^71^ also identified the potential value of some of the same “cold tolerant” crops as we included in this study (i.e., wheat and canola). Nevertheless, this US work also included potatoes, which we excluded owing to the frost sensitivity of potato foliage (Table 2). The US work also considered the potential benefits of expansion of seaweed and aquaculture (to produce additional food for people and animal feed), crop relocation, greenhouse construction, and investing in promising industrial resilient foods. The latter includes repurposing breweries and pulp and paper mills to produce food via the conversion of lignocellulosic biomass such as plant residues, leaves, and wood into edible sugars. Another such approach is the industrial transformation of natural gas/biogas into protein. If such foods were unacceptable as food for people (albeit the protein foods likely bearing some similarity to the current food “Quorn”), then they could be used to make poultry feed for egg production. All these may warrant more investigation in the New Zealand setting, and indeed there is ongoing research on expanding seaweed production^72^. Research in these and other areas could also identify the ability of New Zealand to feed an expanded population from any refugee arrivals.

### Study strengths and limitations

This study is the first of its kind (to our knowledge) to perform this type of optimization analysis for frost resistant food crop production for nuclear war scenarios. It is, however, simplistic in numerous ways as outlined below.

- There are still major uncertainties around the scale of nuclear winter impacts from possible nuclear war scenarios and the modeling uses various simplifying assumptions. For example, the estimates we used from Xia et al.^16^ for New Zealand were for selected major crops and marine fish and we extrapolated from these to across-the-board reductions in food productivity for some of the nuclear winter impact scenarios. Although the model by Xia et al. considered impacts on “surface air temperature, precipitation and downward direct and diffuse solar radiation”, it did not consider potential damage to agriculture from increased ultra-violet light after a nuclear war^73^.
- Our crop yield and nutrition data were not entirely New Zealand specific and some of the ranges in productivity were large (e.g., the reported carrot crop production in New Zealand varies from 70 to 170 t/ha, Table 3). We also only considered one crop planting per year and yet for some crops (especially vegetables) there could conceivably be two plantings per year in some northern regions if these experienced milder nuclear winter conditions. For some crops we only included the root component of the crop (swedes, sugar beet, and onions), and yet in more desperate circumstances the foliage of these crops could also be made available for human consumption.
- We did not account for food wastage after leaving the farm gate (e.g., in the grain milling process, transport, or at the household level). For example, for fresh vegetables the avoidable wastage at the household level can be notable (e.g., 13% when using the mid-point of three European studies^74^, and 20% for the UK^75^).
- Other than dietary energy and protein, the optimization process did not include other nutrients (e.g., other macronutrients and micronutrients). Similarly, we did not consider issues around compounds that can impede micronutrient uptake (e.g., glucosinolates and phytates in canola meal) that may ideally need additional processing to optimize for human nutrition^48^. These issues should ideally be addressed in future research but may not be particularly serious ones given that in a nuclear winter situation there are likely to be some of the food sources detailed in our Scenario B (i.e., some frost sensitive crops, some livestock products and produce from home and community gardening).
- The objective function used in the optimization was only minimizing the cropping land required. A more ideal one would have been estimated post-war prices at the retail level in cities and towns. However, such prices are particularly hard to estimate for nuclear winter scenarios given that the scarcity of imports could dramatically increase the prices of seeds, fertilizer, pesticides, biodiesel and machinery parts (while the price of labor would drop with the loss of export markets).

### Possible implications for research and planning by government agencies

A key role of government is to ensure that essential needs of the population are met. Therefore, considering our findings and the associated uncertainties, the New Zealand Government could consider conducting or commissioning research on the following:

1. New Zealand-specific climate/weather modeling of nuclear winter scenarios to determine likely growing seasons for key frost resistant crops and the risks of out-of-season frosts (e.g., perhaps building on climate and wheat crop models built for Australia^76^). This could guide expansion of both frost resistant crops (pre-war and/or immediately after), and also determine the scope for *frost sensitive* crops in warmer parts of the country. Uncertainty in such variables as crop yields and wastage could be captured in a probabilistic sensitivity analysis.
2. Identifying levels of imported seed for critical crops and explore the logistics of having a New Zealand-based stockpile of extra seed to cover gaps for a whole growing season. If analysis shows it is logistically possible, then seeds could be collected at the first harvest in a nuclear winter situation, and a self-sustaining system, that is independent of imports, could be established.
3. Identifying all vulnerabilities of the agricultural sector to collapse of global trade and loss of imported commodities. As well as various seed imports, these include imports of some types of fertilizers, pesticides, spare parts for farm machinery, and diesel (the latter being required for harvesting, processing and transportation to food processors/markets). Similarly, the extent to which New Zealand production of critical inputs could be scaled up (e.g., biodiesel from canola crops, and domestic fertilizer production). Consideration could also be given to re-establishing onshore refining capacity for liquid fuels from the oil and gas produced in the Taranaki Region.
4. Identifying crop-specific vulnerabilities to damage/loss of local infrastructure integrity. These include access: to electricity for irrigation; to locally produced biodiesel; and to the railway network for transportation to markets (e.g., assuming that adequate fuel for truck transport might not be available).
5. Identifying the potential of some crops having non-food alternative uses for which farmers might get higher prices – and therefore constrain food supplies. These include the use of grain crops for brewing alcohol and for biodiesel production. The latter is relevant to such frost resistant crops as canola, sugar beet, fodder beet, and lupins. Shortages of imported pharmaceuticals could also mean that some farmers switch to growing relevant source crops (e.g., poppies for morphine production).
6. Identifying the capacity of the government being able to disperse funds to citizens in need, so that they could pay farmers or food retailers. Potential vulnerabilities here are the extent of dependency of financial networks and government systems on computing cloud storage in other countries (which could be destroyed in a nuclear war). A Reserve Bank stockpile of paper money or gold coinage might be worth considering (the latter could also be used to help stabilize the currency and buy liquid fuel from Australia).
7. Identifying the feasibility of the expansion of urban horticulture (e.g., building on international research^77^ and existing New Zealand initiatives such as Māori community gardens^63^), particularly in relation to frost resistant vegetables. Urban heat effects and heat from buildings may offer extra protection to such vegetables from out-of-season frosts.
8. Identifying the feasibility of maintaining grass-fed livestock production on hill country near towns and cities and along railway networks (especially the electrified part of the North Island railway network).
9. Identifying the feasibility of maintaining shipping trade with Australia (e.g., to allow wheat imports to New Zealand) and with Pacific Island nations (e.g., those that export copra, palm oil and fish). This would depend on the complexities of sustaining regional shipping in the absence of functioning global shipping routes and international vessels and New Zealand having enough goods or services to trade in return (or potentially using gold from a strategic reserve that was established in New Zealand in the pre-war period).
10. Identifying the value of New Zealand Government investment into breeding more frost resistant cultivars for key crops (for current use and/or for seed stockpiles). These cultivars could increase survival during extra cold winters (as per crops such as winter wheat), but also to freeze–thaw cycles in autumn and spring (see *Introduction*).

If New Zealand Governments and society failed to conduct such research and make adequate preparations, the level of government intervention to ensure food security might have to involve relatively severe measures. These could involve rationing of key inputs to just the most efficient forms of agriculture (e.g., diesel/biodiesel supplies, fertilizer, pesticides, and spare parts for farm machinery). There could be similarities to the requirement for petrol rationing in New Zealand in World War Two (WW2)^78^. The government might also have to prohibit the use of human-edible crops being fed directly to livestock so it can be diverted to feed people. The use of grains being used for alcohol manufacture (e.g., barley used for malting beer), might also need to be suspended for a period. If there were shortages of food supplies, then food rationing for citizens might be needed, as in WW2 in New Zealand^78^.

### Relevance to other sunlight-blocking catastrophes

These findings may also have some relevance to other potential sunlight-blocking catastrophes such as large magnitude volcanic eruptions^79^, and large asteroid/comet impacts^80^. For example, the volcanic eruption of Mt Tambora in Indonesia in 1815, cooled global land temperatures in 1816 by an estimated − 1.9 °C (± 0.2 °C)^81^, and contributed to famines far away from Indonesia (e.g., parts of Europe, India and China^27^). Eruptions of the Tambora scale and larger (magnitudes 7 and 8 + on the volcanic explosivity index), occur around 1.6 times per 1000 years^82^, (equivalent to around a one in six chance per century^83^).

## Conclusions

At current production levels, frost resistant food crops could not feed all New Zealand citizens following a nuclear war. There is a need for the New Zealand Government to conduct a detailed pre-war analysis on how these shortfalls are best addressed. For example, by: increased pre-war production of these crops and/or post-war scalability; growing enough *frost sensitive* crops (i.e., in greenhouses or the warmest parts of the country); and/or ensuring continuing production of food derived from livestock fed on frost resistant grasses.

## Supplementary Information

Supplementary Information.

## Data Availability

All data generated or analysed during this study are included in this published article and in a supplementary information file.
